# Chemogenomic Study of Carboplatin in *Saccharomyces cerevisiae*: Inhibition of the NEDDylation Process Overcomes Cellular Resistance Mediated by HuR and Cullin Proteins

**DOI:** 10.1371/journal.pone.0145377

**Published:** 2015-12-21

**Authors:** Graziele Fonseca de Sousa, Maira de Assis Lima, Débora Fernandes Custodio, Vanessa Morais Freitas, Gisele Monteiro

**Affiliations:** 1 Departamento de Tecnologia Bioquímico-Farmacêutica, Faculdade de Ciências Farmacêuticas, Universidade de São Paulo, São Paulo–USP, Brazil; 2 Departamento de Biologia Celular e do Desenvolvimento, Instituto de Ciências Biomédicas, Universidade de São Paulo, São Paulo–USP, Brazil; University of Tokyo, JAPAN

## Abstract

The use of carboplatin in cancer chemotherapy is limited by the emergence of drug resistance. To understand the molecular basis for this resistance, a chemogenomic screen was performed in 53 yeast mutants that had previously presented strong sensitivity to this widely used anticancer agent. Thirty-four mutants were responsive to carboplatin, and from these, 21 genes were selected for further studies because they have human homologues. Sixty percent of these yeast genes possessed human homologues which encoded proteins that interact with cullin scaffolds of ubiquitin ligases, or whose mRNA are under the regulation of Human antigen R (HuR) protein. Both HuR and cullin proteins are regulated through NEDDylation post-translational modification, and so our results indicate that inhibition of this process should sensitise resistant tumour cells to carboplatin. We showed that treatment of a tumour cell line with MLN4924, a NEDDylation inhibitor, overcame the resistance to carboplatin. Our data suggest that inhibition of NEDDylation may be a useful strategy to resensitise tumour cells in patients that have acquired carboplatin resistance.

## Introduction

Platinum-based drugs, such as cisplatin, oxaliplatin and carboplatin, are used in the treatment of many of the more aggressive and hard to treat cancers, including those of the lung (non-small and small cell cancers), breast, bowel, oesophagus, testes, cervix and ovaries, as well as non-Hodgkin’s lymphoma. Carboplatin is the least toxic of the platinum-based drugs, but like all promoters of DNA damage, its effectiveness reduces on a patient-by-patient basis over multiple chemotherapy cycles due to the emergence of resistance. In this context, understanding the molecular basis of drug resistance could lead to the clinical ability to overcome acquired resistance in tumours using a resensitising molecule, resulting in an improved treatment outcome.

However, studying these processes directly in human cells is expensive and difficult, and reducing the number of animals used in pre-clinical tests is increasingly demanded, requiring previous directional studies to provide a smaller number of targets to be validated *in vivo* and finally in humans. Yeast chemogenomic studies have been of considerable value in identifying biomarkers indicative of possible human cell responses upon drug exposure [1]. Hillenmeyer *et al*. (2008) [2] published a “yeast genomic portrait”, where more than 1100 chemogenomic assays were used to test the fitness of yeast knockout collections (YKO) when exposed to compound libraries of small molecules, including carboplatin. The entire dataset is in the public domain (http://chemogenomics.med.utoronto.ca/fitdb/fitdb.cgi), and so we used this large-scale fitness assay as the preliminary data to select 53 mutant strains that showed the strongest phenotype in response to exposure to the lowest tested concentration of carboplatin.

The growth behaviour and viability of these cells when challenged with carboplatin was analysed in detail. Thirty-four yeast strains (S1 Table) were responsive in the first 24 hours of exposure and were classified in relation to the response of wild-type strain as sensitive or resistant. Among the yeast genes found, 21 possessed human homologues, and these were selected for further systematic analyses. Using bioinformatics tools, it was found 15 human homologue genes whose mRNAs are under the regulation of Human antigen R (HuR; also known as ELAVL1) protein. Additionally, 10 human homologue gene products interact with cullin 1, 2 or 3. The connection between HuR and cullin proteins in the cellular response to carboplatin could be the NEDDylation pathway, since both are regulated by it. NEDDylation is a post-translational modification that modulates the target’s activity/function, through the attachment of the NEDD8 molecule. In agreement with this hypothesis, several cancers have shown increased NEDD8 expression and over-activated cullin-RING ligases (CRLs), whose activation is dependent on NEDD8 attachment, suggesting that NEDDylation is closely related to tumorigenesis [3]. We tested this hypothesis by treating an ovarian tumour cell line with MLN4924 –a NEDD8-activating enzyme (NAE) inhibitor–and carboplatin. Our results suggest that the inhibition of NEDDylation could be a promising way to overcome carboplatin resistance.

## Materials and Methods

### Source and maintenance of yeast cells

Wild-type *Saccharomyces cerevisiae* strain BY4741 (*MATa his3Δ1 leu2Δ0 met15Δ0 ura3Δ0*) and the yeast *Knockout* (YKO) *deletion collection* (Invitrogen, Carlsbad, CA, USA) of non-essential gene knockouts, which is isogenic to BY4741, were used in this study. Cells were grown in YPD medium (2% w/v glucose, 2% w/v peptone, 1% w/v yeast extract, 2% w/v agar added in the case of solid media) or SD complete synthetic medium (0.67% w/v yeast nitrogen base, 0.12% w/v dropout mixture of amino acids, excluding leucine, tryptophan and histidine, 2% w/v glucose, 60 μg/ml leucine, 20 μg/ml histidine, 40 μg/ml tryptophan, 20 μg/ml uracil, pH 6.5). Carboplatin was purchased from Sigma-Aldrich (St. Louis, MO, USA) and MLN4924 from Merck (Merck-Millipore, Billerica, MA, USA).

### Criteria of mutant selection in Hillenmeyer’s dataset

Using the data from the “yeast genomic portrait” (http://chemogenomics.med.utoronto.ca/fitdb/fitdb.cgi), the yeast mutant strains that presented a fitness defect when exposed to 250 μM carboplatin (the lowest dose tested) were selected [2]. This criterion was imposed so that the most drug-sensitive strains would be chosen. The authors considered a significant fitness defect when the P-value was lower than 0.01, which means a confidence of 99%. After screening the dataset using these parameters, 53 mutant strains in candidate genes were isolated whose deletions would confer the highest response to carboplatin exposure (S1 Table). The mutant strains selected were organised individually in a 96-well plate. It should be noted that the present study was performed with the YKO haploid collection.

### Treatment of yeast cells with carboplatin

The yeast mutant cells were grown in SD medium supplemented with 200 μg/ml geneticin G-418 (USB Affymetrix Brand, Santa Clara, CA, USA) at 30°C for 72 hours with 200 rpm shaking. Cells were diluted to an initial optic density of OD_600nm_ = 0.0625 in 96-well plates in SD medium containing or not (control) 10 mM carboplatin. This high drug concentration was chosen because we aimed for a loss of viability and not only a fitness defect. The wild-type strain was grown under the same conditions, but in the absence of G-418. The growth ratio of cells was determined spectrophotometrically using a SpectraMax plate reader at 600 nm (Molecular Devices, Sunnyvale, CA, USA), collecting data every 2 hours for 24 hours. The growth ratios (increase or decrease due to carboplatin exposure) were calculated by obtaining the slope of the OD_600nm_ value versus time curve, during the logarithmic phase, in comparison to the growth ratio of YKO mutant cells and wild-type cells in the absence of carboplatin. The viability of cells after the experiment was determined by sub-culturing 5 μl of each yeast sample for 2 days at 30°C on YPD agar. All the measurements were performed in biological triplicates, and the data were examined using analysis of variance with multiple comparisons (ANOVA) with the statistical software Prism version 6.05 for Windows (GraphPad Software, La Jolla, CA, USA).

### Bioinformatics analyses

The selected genes were analysed using the *Saccharomyces* Genome Database (SGD) tool, *YeastMine*–Batch Analysis (http://yeastmine.yeastgenome.org/yeastmine/). Using the “Analyse” tab, the list of all genes whose deletion caused some response to carboplatin was inputted. Through the “Homologues” tab, it was possible to retrieve human homologue(s) of yeast gene(s) and any of their associated OMIM (*Online Mendelian Inheritance in Man*) disease phenotypes. Finally, the list of human homologue genes involved in resistance to carboplatin and (separately) the genes involved in sensitivity to carboplatin were inputted into the *MetabolicMine* tool (http://www.metabolicmine.org/). Each tool presents an internal statistical measurement that is cited when necessary. When the data were compiled and analysed further, a one-sample *t-*test was used (GraphPad Software). Gene Expression Omnibus (GEO) and Oncomine datasets were used to confirm the differential expression of mRNAs thought to be stabilised by HuR in ovarian cancer in human patients. The analyses were performed by comparing Normal x Cancer, specifically for ovarian cancer. Only patients whose mRNA levels presented an increase of ≥ 40% compared to normal tissue levels were considered overexpressers. In addition, the Cancer Genome Atlas study (TCGA 2) present on Oncomine was analysed to observe differences in the DNA copy number of the genes. The Human Protein Atlas and Database of Differentially Expressed Proteins in Human Cancer (dbDEPC 2.0) were used to analyse the expression of cullins, NEDD8, NAE1 and UBE2M proteins.

### Ovarian tumour cell line challenge with carboplatin

The cell line ES-2 was developed from a clear-cell carcinoma of the ovary; it has a fusiform morphology. Cells were obtained from the collection of the Rio de Janeiro Cell Bank. ES-2 cells were cultured in Dulbecco’s Modified Eagle’s Medium-F12 (DMEM-F12, Sigma Chemical Co., St. Louis, MO, USA) supplemented with 10% foetal bovine serum (FBS; Cultilab, Campinas, SP, Brazil). Cells were maintained in 75-cm^2^ flasks in a humidified atmosphere of 5% CO_2_ at 37°C. The ES-2 cell line was exposed to 50 μM carboplatin once a week for two consecutive weeks to create the chemoresistant phenotype.

### Treatment of the tumour cell line with carboplatin and MLN4924

ES-2 cells presenting a chemoresistant phenotype or ES-2 control cells (untreated) were plated in 24-well plates (2x10^5^ cells/well). Cells were cultured in DMEM-F12 supplemented with 10% charcoal-stripped FBS for 24 hours. After that, the cells were treated with 500 μM carboplatin (Sigma), 10 nM MLN4924 (Merck) or both substances diluted in phenol red-free DMEM/F12 with 10% FBS for 24 hours. Cells without treatment with carboplatin or MLN4924 served as the control. After 24 hours, the cells were released from the plate with trypsin and counted in a Neubauer chamber; the results are presented as the percentage of viable cells. All the measurements were performed in biological triplicates, and the data were analysed using ANOVA with multiple comparisons with the statistical software GraphPad Prism version 6.05.

## Results and Discussion

**Fig 1:**
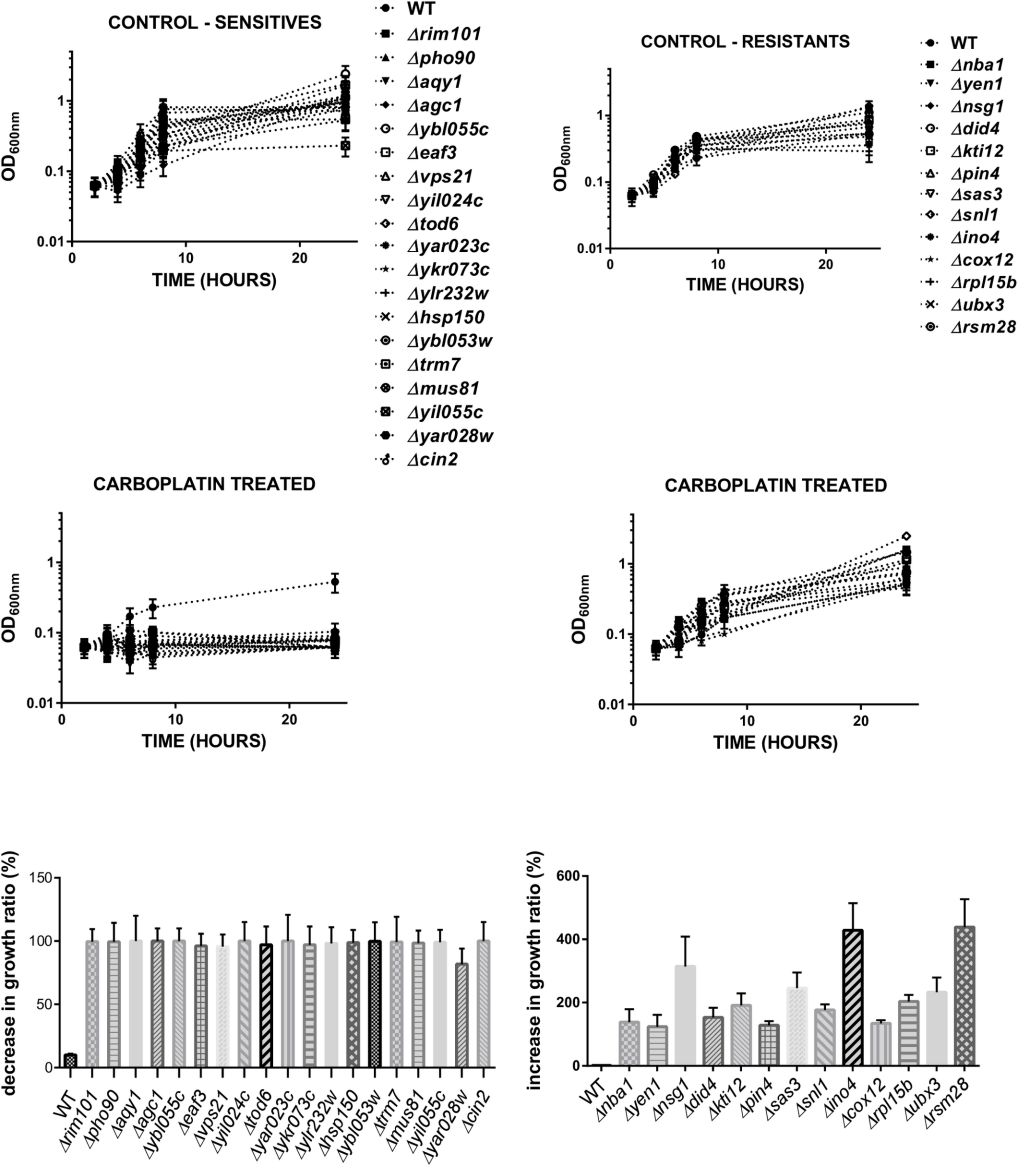
Growth rates of the yeast mutants in response to carboplatin exposure. The cells were thawed and grown in synthetic dextrose minimum media (SD) at 30°C for 72 hours in the presence of the antibiotic geneticin (G418), except for the wild-type strain. These reactivated cells were then normalised to an initial OD_600nm_ of 0.0625 in fresh SD media in the presence (carboplatin treated) or absence (control) of carboplatin 10 mM and the growth rates were spectrophotometrically analysed during 24 hours (see Material and Methods). These values were quantitatively represented as the percent decrease (sensitive) or increase (resistant) in the growth ratio in relation to the untreated cell response. The results are presented as mean and SD of three independent experiments, analysed by one-way ANOVA with multiple comparisons in relation to the wild-type strain, p = 0.0001, using GraphPad software.

**Fig 2:**
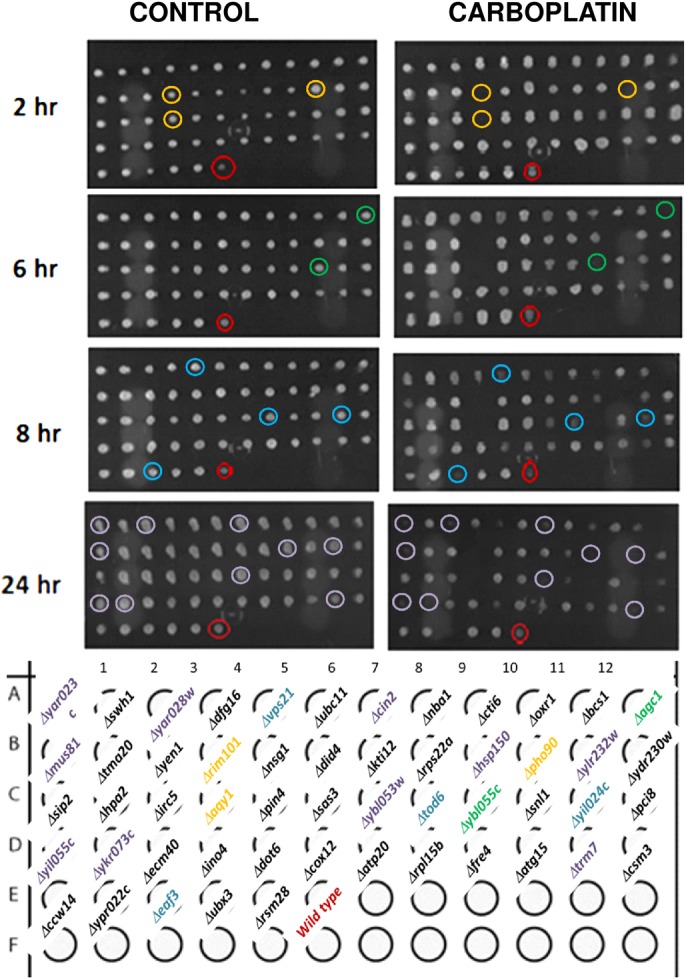
Cell viability analyses during different hours of carboplatin exposure. At the indicated times, aliquots of the yeast mutant cultures in the presence (carboplatin) or absence (control), whose growth are shown in Fig 1, were withdrawn and dripped with a replicator onto a YPD plate, which was incubated at 30°C for 2 days and photographed. These images are representative of at least three biological replicates.

We tested the growth ratio and cell viability of the 53 selected yeast mutants and observed that 19 were sensitive and 15 resistant after 24 hours of exposure to 10 mM carboplatin (Fig 1). The increase or decrease in the growth ratio caused by carboplatin exposure of each individual strain is quantitatively shown in the lower panels (Fig 1). The difference in the phenotypes observed in this study compared to Hillenmeyer’s data–since according to the fitness results, all the strains should be highly sensitive–may be due to variations in the type of yeast collection used, as well as the time and dose of carboplatin exposure applied. The final classification as sensitive or resistant mutant cells was also based on the viability loss, which was measured by the ability of the each individual yeast strain to form colonies in fresh rich solid medium (YPD) after the different periods of drug exposure during growth (Fig 2). Table 1 shows the 34 yeast genes whose deletion caused some phenotypic response to carboplatin exposure, their standard name and the presence or absence of known human homologues (Table 1).

**Table 1:** Identification of the yeast set selected.

Systematic Name	Standard Name	Human Homolog	Systematic Name	Standard Name	Human Homolog
*YAR023C*		No	*YDL091C*	*UBX3*	Yes
*YAR028W*		No	*YDR386W*	*MUS81*	Yes
*YBL051C*	*PIN4*	Yes	*YDR494W*	*RSM28*	No
*YBL052C*	*SAS3*	Yes	*YJL159W*	*HSP150*	No
*YBL053W*		No	*YJL198W*	*PHO90*	Yes
*YBL054W*	*TOD6*	No	*YLR038C*	*COX12*	Yes
*YBL055C*		Yes	*YLR232W*		No
*YBR061C*	*TRM7*	Yes	*YMR121C*	*RPL15B*	Yes
*YER041W*	*YEN1*	Yes	*YOL070C*	*NBA1*	No
*YHL027W*	*RIM101*	Yes	*YOL108C*	*INO4*	No
*YHR133C*	*NSG1*	Yes	*YOR089C*	*VPS21*	Yes
*YIL024C*		No	*YOR339C*	*UBC11*	Yes
*YIL046W*	*MET30*	Yes	*YPL196W*	*OXR1*	Yes
*YIL055C*		No	*YPL241C*	*CIN2*	No
*YKL002W*	*DID4*	Yes	*YPR021C*	*AGC1*	Yes
*YKL110C*	*KTI12*	Yes	*YPR023C*	*EAF3*	Yes
*YKR073C*		No	*YPR192W*	*AQY1*	Yes

Yeast set selected for further characterisation in this work, previously involved in the cellular response to carboplatin, according to the fitness defect showed in the presence of carboplatin. Total number of genes analysed = 34; genes that possess human homologs = 21 (62%); genes that do not possess human homologs = 13 (38%).

Because our aim was to investigate the human drug response, of the 34 yeast strains, we selected 21 candidate genes that presented human homologues (Table 1). We next used the SGD tool (http://yeastmine.yeastgenome.org/yeastmine/) to correlate human diseases to the set of yeast genes responsive to carboplatin and found two human homologue genes that were previously associated with cancer: Kruppel-like factor 6 (KLF6, selected because it is one of the homologues of the yeast gene *RIM101*) and Excision Repair Cross Complementing Group 5 (ERCC5, selected because it is the homologue of the yeast gene *YEN1*) (Table 2). The *Δrim101* (gene *YHL027W/RIM101*) mutant was one of the most sensitive strains observed in this study and lost its viability after only 2 hours of exposure to carboplatin (Fig 2). The KLF6 human homologue of the yeast gene *RIM101* has been implicated in gastric and prostate cancer, tumours that are usually treated with carboplatin. The second yeast mutant possessed the *YEN1* deletion and was resistant to carboplatin. This result is in agreement with the suggestion that the correspondent human homologue ERCC5 is involved in DNA repair and can be used as a biomarker of survival in patients with ovarian cancer treated with carboplatin [4]. Also, specific ERCC5 promoter polymorphisms have been shown to increase the platinum chemotherapy response in patients with advanced non-small-cell lung cancer (NSCLC) [5]. These data help to validate our results obtained using yeast as a primary model cell to identify human genes involved in cancer chemoresistance.

**Table 2:** Human diseases related to genes involved in the cellular response to carboplatin.

Yeast standard name	Human homolog standard name	Diseases	Datasets
*AGC1*	SLC25A12	Hypomyelination, global cerebral	Ensembl, Panther, TreeFam
*AGC1*	SLC25A13	Citrullinemia, typeII, neonatal-onset and adult onset—CTLN2	Ensembl, Panther, TreeFam
*AGC1*	SLC25A22	Epileptic encephalopathy early infantile - 3EIEE3	Ensembl, Panther, TreeFam
*AQY1*	AQP1	Blood group–Colton CO	Ensembl, Panther
*AQY1*	AQP2	Diabetes insipidus, Nephrogenic, autosomal	Ensembl, Panther, TreeFam
*AQY1*	AQP3	Gill blood group	Panther
*AQY1*	AQP5	Palmoplantar keratoderma, Bothnian type PPKB	HomoloGene, TreeFam
*AQY1*	AQP7	Glycerol quantitative trait locus—GLYCQTL	Panther
*AQY1*	MIP	Cataract 15, Multiple types—CTRCT15	Ensembl, Panther, TreeFam
*DID4*	CHMP2B	Frontotemporal dementia, chromosome 3-linked FTD3 and amyotrophic lateral sclerosis 17 ALS17	Ensembl
*RIM101*	KLF1	Blood group- Lutheran inhibitor INLU; anemia, dyserythropoietic congenital, type IV—CDAN4; and fetal hemoglobin quantitative trait locus 6—HBFQTL6	Panther
***RIM101***	**KLF6**	**Gastric cancer, intestinal included and prostate cancer**	Panther
*RIM101*	SP7	Osteogenesis imperfecta, type XII—OI12	Panther
*RPL15B*	RPL15	Diamond–Blackfan anemia 12 DBA12	Ensembl, HomoloGene, Panther, TreeFam
*TRM7*	FTSJ1	Mental retardation, X-linked 9 -MRX9	Ensembl, HomoloGene, Panther
***YEN1***	**ERCC5**	**Xeroderma pigmentosum, complement group GXP G**	Panther

The analysis was performed using the tool YeastMine: Advanced Search–HOMOLOGY—Yeast gene→ OMIM human homolog(s) → OMIM Disease Phenotype(s) in the SGD site www.yeastgenome.org.

We continued the analysis of the 21 yeast genes and observed that they possess 88 homologous genes in humans (Table 3): 57 human homologues to yeast genes related to resistance and 31 human homologues to yeast genes related to sensitivity. It is important to note that yeast genes whose disruptions led to sensitivity are probably responsible for resistance to carboplatin (nine genes identified: *YBL055C; TRM7; MUS81; RIM101; PHO90; VPS21; AGC1; AQY1*), and yeast genes whose disruptions led to resistance may be related to sensitivity to this drug (12 genes identified: *PIN4; SAS3; UBX3; YEN1; NSG1; MET30; DID4; KTI12; COX12; RPL15; UBC11; OXR1*). This nomenclature will be used hereafter, including for classifying human homologues. Human homologue genes were analysed regarding their chromosome localisation, metabolic pathway, molecular function and interactions using the *MetabolicMine* tool (http://www.metabolicmine.org/).

**Table 3:** Human homologues identified in the study of yeast carboplatin response.

Yeast gene involved in resistance	Human homolog	Yeast gene involved in resistance	Human homolog	Yeast gene involved in sensitivity	Human homolog
*AQY1*	AQP1	*AQY1*	MIP	*MET30*	BTRC
*AQY1*	AQP2	*EAF3*	MORF4L1	*DID4*	CHMP2A
*AQY1*	AQP3	*EAF3*	MORF4L2	*DID4*	CHMP2B
*AQY1*	AQP4	*EAF3*	MSL3	*COX12*	COX6B1
*AQY1*	AQP5	*MUS81*	MUS81	*COX12*	COX6B2
*AQY1*	AQP7	*VPS21*	RAB17	*YEN1*	ERCC5
*AQY1*	AQP8	*VPS21*	RAB20	*UBX3*	FAF1
*AQY1*	AQP9	*VPS21*	RAB22A	*UBX3*	FAF2
*AQY1*	AQP10	*VPS21*	RAB31	*MET30*	FBXW11
*AQY1*	FAM188B	*VPS21*	RAB5A	*MET30*	FBXW2
*TRM7*	FTSJ1	*VPS21*	RAB5B	*MET30*	FBXW7
*TRM7*	FTSJ2	*VPS21*	RAB5C	*NSG1*	INSIG1
*RIM101*	KLF1	*PHO90*	SLC13A1	*NSG1*	INSIG2
*RIM101*	KLF2	*PHO90*	SLC13A2	*SAS3*	KAT5
*RIM101*	KLF3	*PHO90*	SLC13A3	*SAS3*	KAT7
*RIM101*	KLF4	*PHO90*	SLC13A4	*KTI12*	KTI12
*RIM101*	KLF5	*PHO90*	SLC13A5	*OXR1*	NCOA7
*RIM101*	KLF6	*AGC1*	SLC25A12	*OXR1*	OXR1
*RIM101*	KLF7	*AGC1*	SLC25A13	*PIN4*	PABPC1
*RIM101*	KLF8	*AGC1*	SLC25A18	*PIN4*	PABPC1L
*RIM101*	KLF9	*AGC1*	SLC25A22	*PIN4*	PABPC4
*RIM101*	KLF10	*AGC1*	SLC25A44	*PIN4*	PABPC4L
*RIM101*	KLF11	*RIM101*	SP5	*PIN4*	PABPC5
*RIM101*	KLF12	*RIM101*	SP6	*RPL15B*	RPL15
*RIM101*	KLF13	*RIM101*	SP7	*PIN4*	SYNCRIP
*RIM101*	KLF14	*YBL055C*	TATDN1	*OXR1*	TLDC2
*RIM101*	KLF15	*YBL055C*	TATDN2	*MET30*	TRAF7
*RIM101*	KLF16	*YBL055C*	TATDN3	*UBC11*	UBE2C
*RIM101*	KLF17			*UBC11*	UBE2U

**Fig 3:**
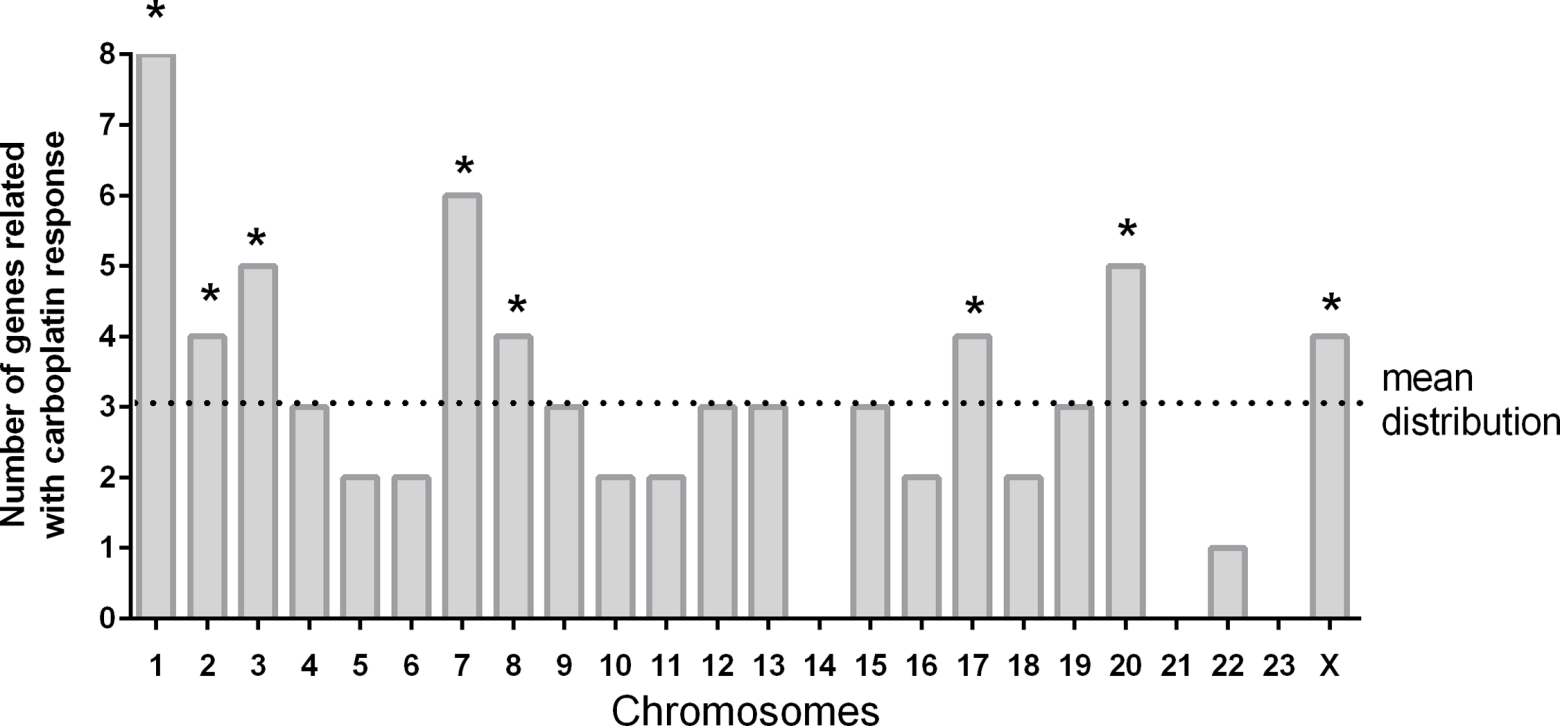
Chromosome distribution of human homologues predicted to be involved in carboplatin response. The graph was constructed using human chromosome localisation (retrieved from *metabolicmine* analysis) of each gene that presented different yeast gene deletions that caused the phenotype, i.e. chromosome 1 possesses at least eight different human genes corresponding to eight different yeast mutant strains. The arithmetic mean distribution of genes was calculated by the sum of total human genes that possess different yeast homologues divided by the number of chromosomes. The mean distribution was 2.958 genes per chromosome. We calculated the chromosomes that presented an accumulation of genes responsive to carboplatin considering statistical significance in relation to the mean of p = 0.0047 using a one-sample *t*-test.

The chromosome distribution of human homologues of genes involved in the carboplatin response is shown in Fig 3. We calculated the mean distribution of genes involved in the carboplatin response in each chromosome. The number of genes presented on the y-axis corresponds to the number of different mutant yeast strains related to the carboplatin response that possess at least one human homologue in the chromosome depicted on the x-axis. The mean distribution of genes related to the carboplatin response was 2.96 genes in each chromosome, taking into account that in some chromosomes the frequency found was zero (i.e. chromosomes 14, 21 and 23). There was a significantly higher frequency of these homologues on chromosomes 1, 2, 3, 7, 8, 17, 20 and X using one-sample *t-*test statistical analysis. This result is in good agreement with previous studies characterising the high-resolution profiling of carboplatin resistance in ovarian cancer cells [6, 7]. In these reports, the authors associated chromosomes 1, 8, 17 and X with the carboplatin response in ovarian cancer. Interestingly, resistance to carboplatin was mainly associated with chromosome 1, and our results show that this chromosome presented the highest number of genes related to the carboplatin response. In addition, genes on human chromosome 1 have previously been associated with the tumour response to platinum-based drug treatment. For example, genes on chromosome 1 have been related to cisplatin resistance in a human neuroblastoma and to sensitivity in human lymphoblastoid cell lines [8, 9]. This supports the ability of yeast cells to indicate good molecular candidates in the characterisation of the cellular response to carboplatin.

The human homologues of yeast carboplatin-response genes were next annotated for biological and molecular functions (Tables 4 and 5). Table 4 shows the genes that were most likely to be involved in the cellular resistance to carboplatin. The main biological process associated by the Gene Ontology term was transport (29 human homologue genes, derived from the following yeast mutant strains: *Δaqy1*; *Δrim101; Δvps21; Δagc1; Δpho90*). The main biochemical pathway found 11 genes associated with transmembrane transport of small molecules. The main molecular function of 16 genes was substrate-specific transmembrane transporter activity. Not surprisingly, these data suggest that cell resistance to carboplatin is related to import/export of the drug, which is in agreement with a review of the literature on the effects of ion channels and aquaporin expression in ovarian cancer [10]. Xuejun *et al*. [11] demonstrated that when the ovarian cancer cell line SKOV3 is treated with mercuric chloride, which blocks aquaporins, the cells become more sensitive to cisplatin, thereby implicating aquaporins in resistance to platinum-based drugs, similarly to the results observed here (Table 4).

**Table 4:** Metabolic pathways of human homologue genes predicted to be involved in the cellular resistance to carboplatin exposure.

Biological Process		
GO term	P value	MATCHES
transport [GO:0006810]	0.026101	AQP1; AQP2; AQP3; AQP4; AQP5; AQP7; AQP8; AQP9; AQP10; MIP; SLC25A12; SLC25A13; SLC25A22; RAB5A; RAB5B; RAB5C; RAB17; RAB20; RAB22A; RAB31; KLF13; KLF14; KLF15; KLF16; SLC13A1; SLC13A2; SLC13A3; SLC13A4; SLC13A5
Pathway Enrichment		
Pathway	P value	MATCHES
Transmembrane transport of small molecules	0.003694	AQP1; AQP2; AQP3; AQP4; AQP7; AQP9; SLC13A1; SLC13A2; SLC13A3; SLC13A4; SLC13A5
Molecular Function		
substrate-specific transmembrane transporter activity [GO:0022891]	1.799845e-4	AQP1; AQP2; AQP3; AQP4; AQP5; AQP7; AQP8; AQP9; MIP; SLC13A1; SLC13A3; SLC13A4; SLC13A5; SLC25A12; SLC25A13; SLC25A22
Interactions		
Physical interaction	ELAVL1 protein	AQP3; KLF2; KLF5; KLF9; KLF11; MORF4L1; RAB5A; RAB5B; RAB22A; RAB31; SLC25A44

Source *MetabolicMine*. The P-value was calculated using the Hypergeometric distribution.

**Table 5:** Metabolic pathways of human homologue genes predicted to be involved in the cellular sensitivity to carboplatin exposure.

Biological Process		
GO term	P value	MATCHES
SREBP signaling pathway [GO:0032933]	0.002740	INSIG1; INSIG2; FBXW7
cellular macromolecule catabolic process [GO:0044265]	0.005467	BTRC; ERCC5; FAF1; FBXW7; FBXW11; PABPC1; PABPC4; RPL15; UBE2C
Pathway Enrichment		
Pathway	P value	MATCHES
Ubiquitin mediated proteolysis	0.017076	UBE2C; UBE2U; FBXW7; FBXW11; BTRC
Molecular Function		
NO ENRICHMENT		
Interactions		
Physical interaction	CULLIN (S) 1, 2 and 3	SYNCRIP; FAF1; FAF2; FBXW11; FBXW2; FBXW7; PABPC1; PABPC4; RPL15; BTRC
Physical interaction	ELAVL1 protein	UBE2C; FAF2; PABPC1; PABPC4; INSIG1

Source *MetabolicMine*. The P-value was calculated using the Hypergeometric distribution.

Otherwise, Table 5 shows the genes that are most likely to be involved in cell sensitivity to carboplatin. The results indicate that two main biological processes appear to be involved in cell sensitivity: the sterol regulatory element-binding protein (SREBP) signalling pathway (related to the function of three human homologue genes, derived from the following yeast strains: *Δnsg1; Δmet30*) and the cellular macromolecule catabolic process (involving nine human homologue genes, derived from the following yeast mutant strains: *Δpin4; Δmet30; Δyen1; Δubx3; Δrpl15b; Δubc11*). No significant correlation was found with molecular function or regulatory systems. However, the main pathway related to sensitivity was ubiquitin-mediated proteolysis, which is reinforced by the observation that an increased burden of quality-control systems has been associated with aberrant DNA-copy number in yeast and mammals [12], indicating that this pathway is intimately related to tumour biology and, in particular, the response to carboplatin.

Finally, the analyses of physical interactions between molecules provided interesting results; it was intriguing to note that the HuR protein was implicated in both resistance and sensitivity responses to carboplatin exposure (Tables 4 and 5). HuR is a member of the embryonic lethal abnormal vision 1 (ELAV1 or ELAVL1) family and is primarily localised in the nucleus, but under several types of stress is also translocated to the cytoplasm. In the cytoplasm, HuR binds to the 3´-untranslated region of target mRNAs, resulting in improved translation and stability. HuR activation has been observed in response to several cancer-related stressors, such as exposure to gemcitabine, tamoxifen, staurosporine, UV light and actinomycin D, indicating that the HuR protein primarily functions as a cytoprotective mechanism in tumour cells [13]. Recently, Lal *et al*. [14] observed that tumour cell treatment with carboplatin causes HuR translocation from the nucleus to the cytoplasm, showing that this drug modulates HuR activity and suggesting a tumour pro-survival function of HuR during carboplatin exposure.

In addition, a genome-wide screen that used systematic overexpression of 12,200 pooled human ORFs in the human cell line HEK293 was carried out to understand drug mode-of-action and resistance mechanisms. The authors found that four main human ORFs that were overexpressed provide protection against cisplatin cytotoxicity: MAPK kinase 1 interacting protein 1—MAP2K1IP1, monocyte to macrophage differentiation-associated protein—MMD, protein tyrosine phosphatase, non-receptor type 2—PTPN2 and Rhox homeobox family, member 2—RHOXF2 [15]. Analysing the interactors of these ORFs that are implicated in platinum-based drug resistance, by a completely different approach, using the *MetabolicMine* tool, it was found that MAP2K1IP1 and MMD mRNAs interacted with HuR, reinforcing the present hypothesis that HuR mediates platinum-drug resistance.

**Fig 4:**
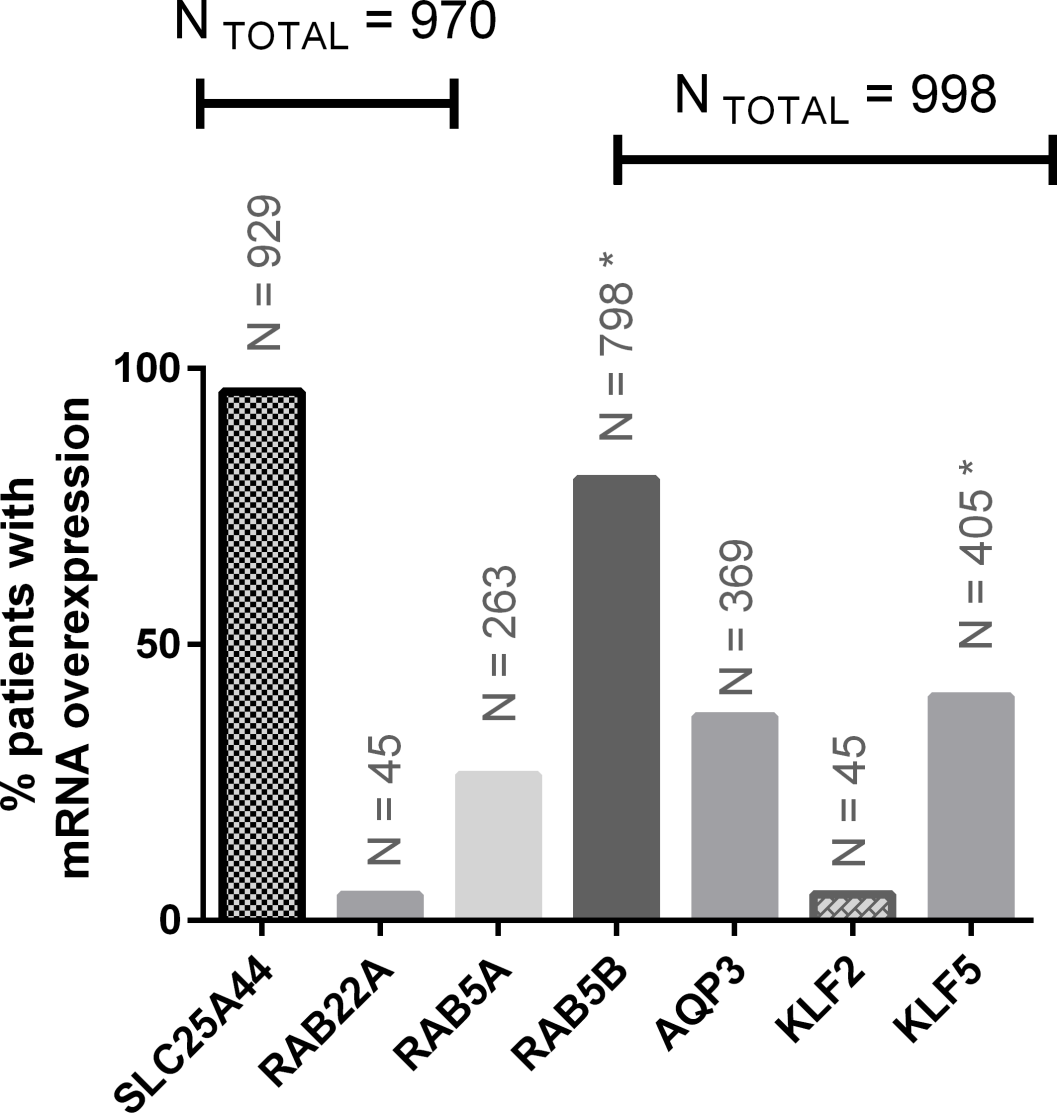
mRNA expression analyses of genes supposed to be stabilised by HuR. The bioinformatics analysis was performed using datasets present in Gene Expression Omnibus and Oncomine. It was considered as overexpression in ovarian cancer just increasing in mRNA level ≥ 40% of expression level in normal tissue (control). The number (N) depicted above each bar is the total of patients that presented overexpression of each gene; the N_TOTAL_ represents the total number of patients studied for each gene.

If HuR is important in ovarian cancer survival and the carboplatin response, the mRNAs shown in Table 4 should be stabilised by HuR and consequently show overexpression in clinical cases of ovarian cancer. To test this hypothesis, we used two large datasets of human gene expression–Gene Expression Omnibus and Oncomine–to analyse the mRNA levels of the SLC25A44, RAB22A, RAB5A, RAB5B, AQP3, KLF2 and KLF5; the mRNAs of these genes have been observed in physical interaction with the HuR protein (S2 Table). Fig 4 shows the percentage of patients with ovarian cancer that presented at least a 40% increase in the mRNA level when compared to the level in the respective normal tissue. It was possible to analyse 970 or 998 patients with ovarian cancer using these datasets; markedly, four genes showed a high frequency of overexpression: SLC25A44, RAB5B, AQP3 and KLF5. RAB5B and KLF5 have been previously characterised as being in the top 4% and 9%, respectively, of genes overexpressed in ovarian cancer [16, 17]. In addition, AQP3, also called aquaglyceroporin-3, has been recently related to the estrogenic response driving cell migration and cell invasion in breast cancer [18]. In a study comparing patients who were either sensitive or chemoresistant to carboplatin, our results were again reinforced with the overexpression found for AQP3, KLF5, RAB5A and RAB22A in chemoresistant patients [19]. Finally, an interesting gene not previously involved in ovarian cancer, SLC25A44, presented a higher frequency of overexpression in 96% of the 970 patients with analysed ovarian tumours. SLC25A44 belongs to the mitochondrial solute carrier family; the gene is localised on chromosome 1 and its molecular function is unknown (UniProtKB—Q96H78).

**Fig 5:**
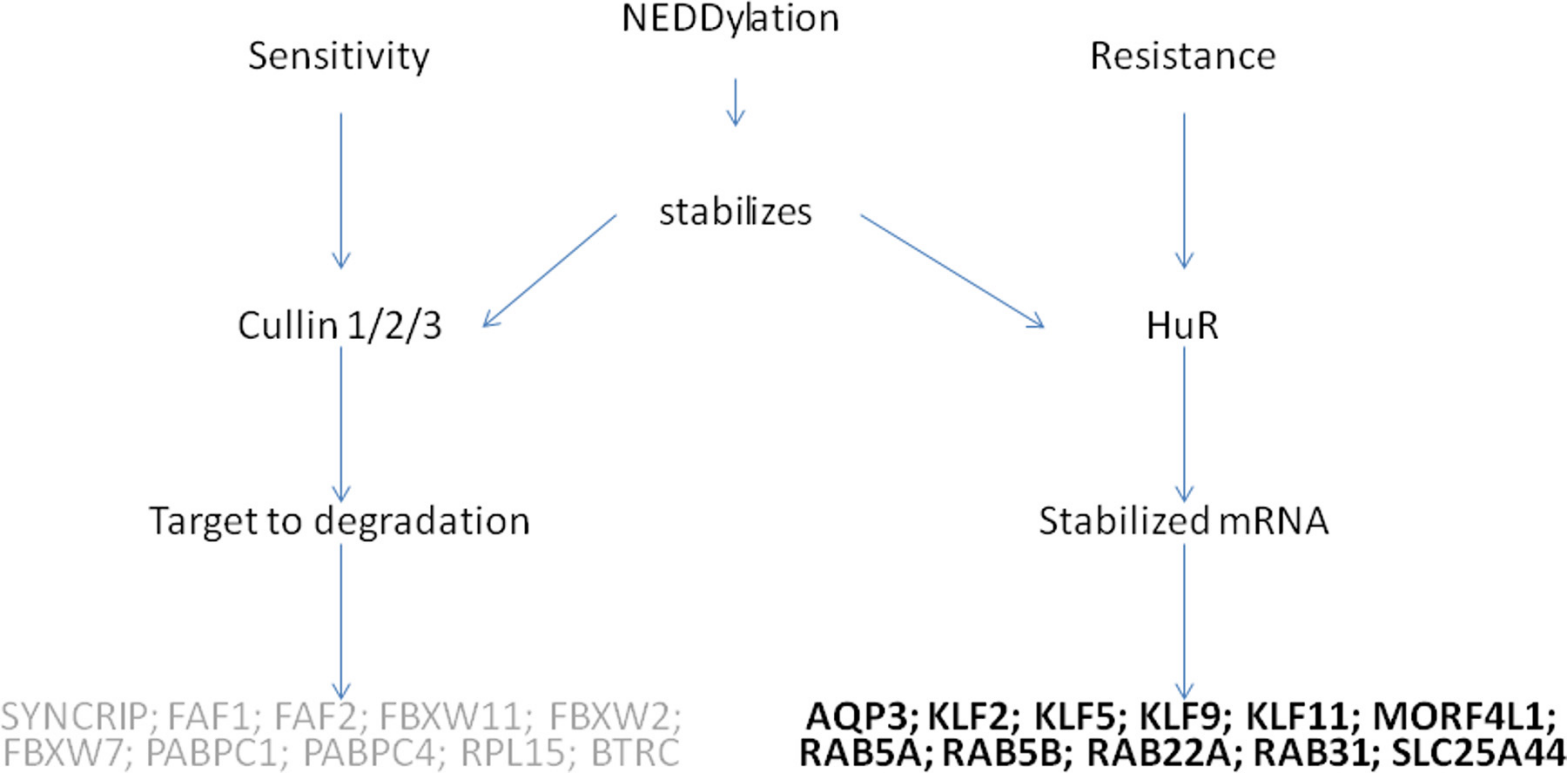
Model of proposed cellular responses involved in carboplatin resistance and susceptibility. A NEDDylation-associated pathway controls nuclear localisation and stabilises HuR and activates cullins 1/2/3 proteins involved in the regulation of 23 human homologues related to the carboplatin response.

Otherwise, ten human homologues of yeast carboplatin-responsive genes were annotated as interacting physically with cullin proteins (Table 5 and S2 Table). Cullins are scaffold proteins that bind to an adaptor protein, a substrate receptor protein and a RING protein, assembling cullin-RING ligases (CRLs)–the largest family of E3 ubiquitin ligases. E3 ubiquitin ligases are important and diverse protein complexes that contribute to the specificity of proteasome-mediated protein degradation [20]. Together, HuR and cullins 1/2/3 regulate 23 different human homologues, which were originated from 12 different yeast genes whose deletion led to a carboplatin response (almost 60% of yeast genes selected; Tables 4 and 5). We propose that, in conjunction, cullins and HuR lead to a loss of proteins responsible for carboplatin sensitivity through ubiquitylation and proteasome-mediated degradation, in addition to increasing the mRNA stabilisation and translation efficiency of proteins related to tumour resistance (Fig 5).

If our hypothesis is correct, the proteins related in Table 5 should be found at decreased levels in ovarian cancer. We first analysed their mRNA expression and no significant difference was observed (data not shown), which was expected since our hypothesis is that cullins (that modulate proteins and not mRNA) were responsible for diminished levels of related proteins. Furthermore, we analysed the mRNA expression of cullins 1, 2, 3, 4A, 4B, 5 and 7. Only cullin 4B and cullin 7 presented overexpression in 77% and 90%, respectively, of 998 patients analysed. This was interesting, but does not explain our results since cullins 1, 2 and 3 found interacting physically with proteins related in Table 5 did not present changes in expression.

**Fig 6:**
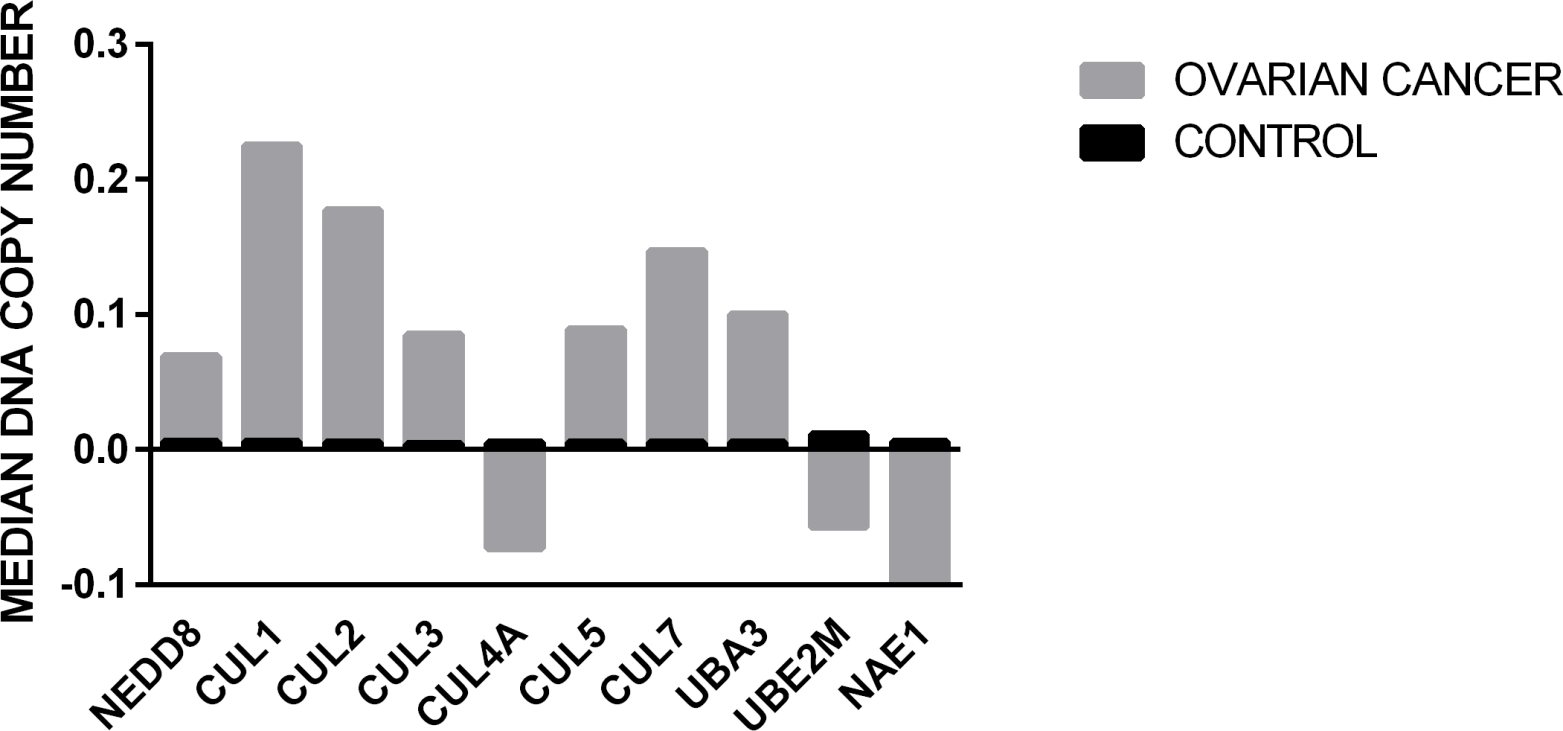
Analysis of DNA copy number aberration of genes involved in NEDDylation pathway in ovarian tumour patients. 1,168 samples were analysed, composed of 431 normal blood, 130 normal ovary tissue and 607 ovarian serous cystadenocarcinoma, present in the Cancer Genome Atlas 2 (TCGA2)- Ovarian Serous Cystadenocarcinoma DNA Copy Number Data. The analysis was performed with Oncomine tool gene analysis using the filters: Cancer x Normal, Cancer type: ovarian. The number depicted in y axis represents the median number of DNA copy in patients analysed. The normal median frequency of DNA copy number was 0.005, almost unchangeable in control samples.

Next, we analysed the TCGA2 dataset (The Cancer Genome Atlas 2, present in Oncomine) to assess changes in DNA copy number. Fig 6 shows that, except for cullin 4A, in the 607 patients with ovarian cancer analysed, the median DNA copy number of cullin encoding genes was increased when compared to normal tissues. In addition, NEDD8 and UBA3 (the E1-NEDD8 activating enzyme that forms a heterodimer with NAE1 [3]) also presented high DNA copy number in ovarian cancer. This is an intriguing fact since these proteins are known to be key components of the NEDDylation pathway [3], and the DNA copy number of NAE1 (the E1-NEDD8 activating enzyme) and UBE2M (E2-NEDD8 ligase) presented a decrease in ovarian cancer patients. However, there are some data which suggest that this decrease in DNA copy number does not necessarily result in less NAE1 and UBE2M in ovarian cancer. Bonome *et al*. [16] characterised UBE2M as being in the top 10% of genes overexpressed in their study on ovarian carcinoma, involving 185 patients. In addition, in the comparison between patients sensitive and chemoresistant to carboplatin (three patients of each group analysed in triplicate), up to a 47% increase in NAE1 expression was observed in ovarian cancer from chemoresistant patients [19].

In addition to increases in DNA copy number and mRNA expression, to provide indirect evidence of increases in encoded protein, we analysed two proteomic databases: the Human Protein Atlas and the Database of Differentially Expressed Proteins in Human Cancer (dbDEPC 2.0). We found that, at the protein level, although proteomic information is much scarcer than mRNA in terms of clinical patient samples available to be analysed, our hypothesis that the NEDDylation pathway is overactive in ovarian cancer was confirmed (Table 6). In the Human Protein Atlas, we found this observation in 12 patient samples and several tumour cell lines; of these, we chose the EFO-21 cell line, an ovarian cystadenocarcinoma, to present the results.

**Table 6:** Comparison of protein expression in ovarian cancer and normal tissue.

PROTEIN EXPRESSION		
	CONTROL	OVARIAN CANCER
CUL1	low expressed	17% tumours presented moderate expression
CUL2	low expressed	67% tumours presented moderate expression
CUL3	moderate expression in stroma	25% tumours presented strong expression
CUL4A	no information	no information
CUL4B	not expressed in stroma	100% tumours presented low expression
UBE2M	not expressed in stroma	strong expression in EFO-21 cell line
NEDD8	moderate expression in stroma	strong expression in EFO-21 cell line
NAE1	moderate expression in stroma	58% tumours presented strong expression
FBXW2	moderate expression	low expressed
PABPC1	not expressed	90% tumours presented low to moderate expression

Cullins are activated by NEDDylation, thereby increasing protein degradation mediated by the ubiquitin-proteasome system (reviewed by Zhao, Morgan and Sun [3]). However, it has only recently been demonstrated that HuR is stabilised by NEDDylation. This stabilisation is mediated by the murine double minute 2 protein (Mdm2) and the levels of these two proteins are correlated in human hepatocellular carcinoma and colon cancers [21]. Therefore, we also suggest that there may be a common feature regulating the resistance or sensitivity of cells to carboplatin–the NEDDylation pathway (Fig 5).

Protein NEDDylation involves the attachment of NEDD8 (an ubiquitin-like molecule) onto a substrate protein. This modification does not lead to degradation itself, but to the modulation of target protein activity and function. In contrast to ubiquitylation, protein NEDDylation is a more restricted event biochemically, with no more than 20 substrates described in the literature [reviewed in 3]. These substrates include key proteins in the regulation of DNA damage responses, such as p53, which is NEDDylated by the same protein that regulates HuR, Mdm2 (reviewed in Zhao, Morgan and Sun [3]).

A small molecule designated MLN4924 is an inhibitor of NEDDylation and is listed as a drug candidate on the NCI/NIH Drug Directory (http://www.cancer.gov/drugdictionary?cdrid=596795). MLN4924 specifically inhibits NEDDylation by acting on NEDD8-activating enzyme (NAE1) and is currently undergoing several Phase 1b clinical trials (http://www.cancer.gov/clinicaltrials/search/results?protocolsearchid=13020760). MLN4924 has also been demonstrated to overcome cisplatin resistance in ovarian tumour cell lines and in a mouse ovarian tumour xenograft model [22]. MLN4924 is synergistic when used in combination with cisplatin; this synergy is dependent on cullin 3, since siRNA knockdown of CUL3 produced a 45% increase in cisplatin cytotoxicity. The authors affirmed that the majority of their results were similar to those with carboplatin [23].

**Fig 7:**
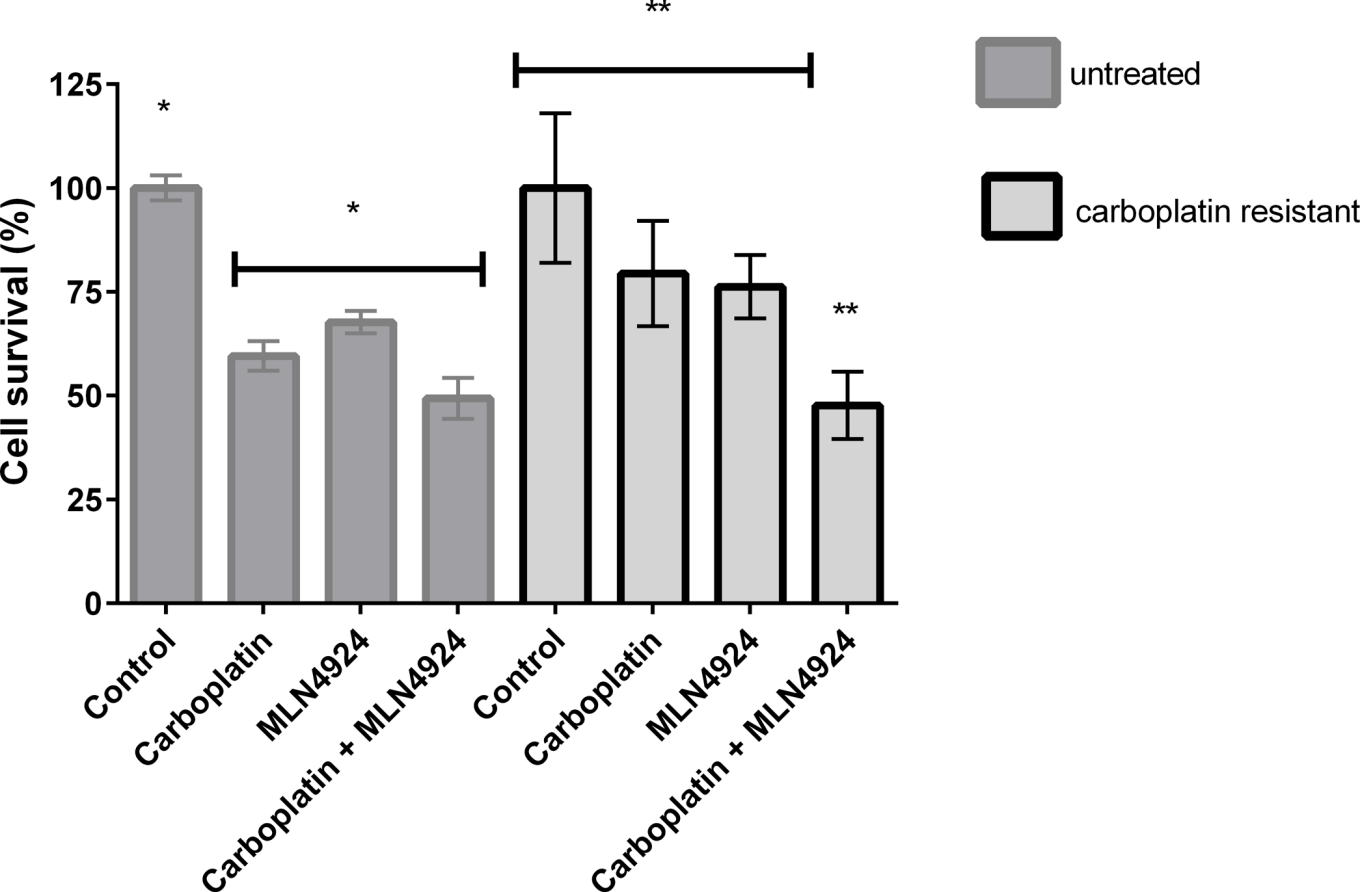
Viability assay of non-treated and chemoresistant ovarian cancer cell lines challenged with carboplatin, NEDDylation inhibitor MLN4924 or both. The ES-2 ovarian cancer cell line was pre-treated with 50 μM carboplatin and the surviving cells were selected as chemoresistant to carboplatin. The chemoresistant and non-treated cells were challenged with 500 μM carboplatin, 10 nM MLN4924 or a combination of both for 24 hours. The results are presented as mean and SD, analysed by one-way ANOVA–multiple comparisons, with statistical significant p = 0.0001, using GraphPad software. The capped lines represent the group of samples that were not statistically different between each other, and the asterisks represent a statistically significant difference between the groups that were compared.

We tested the effect of NEDDylation inhibition with MLN4924 in ES-2 cancer cell lines non-resistant and chemoresistant to carboplatin (Fig 7). In carboplatin non-resistant cells, the viability decrease was similar in cells treated with carboplatin, MLN4924 or a combination of both. The synergy of MLN4924 with cisplatin observed previously [23] was not observed in combination with carboplatin. This can be attributed to the lower dose of MLN4924 (up to 100-fold) or the different culture media used in the present study to obtain the same ratio of tumour cell cytotoxicity. However, although carboplatin chemoresistant cells were not responsive to carboplatin or MLN4924, the combination of drugs was able to overcome resistance, rescuing the sensitivity of these cells to non-resistant levels. These results were also not in agreement with the results with cisplatin resistant cells, since they did not acquire resistance to MLN4924 and the combination with cisplatin was not significantly better than the effect of NEDDylation inhibition alone [23]. Our results suggest that it is not recommended to use a NEDDylation inhibitor at the beginning of patient treatment, because there was no statistical significant improvement in the carboplatin response. However, it was clear that, in the case of tumour cells that acquired resistance during carboplatin treatment, NEDDylation inhibition with MLN4924 rescued the sensitivity of ovarian tumour cells. Interestingly, although the vast majority of literature considers the platinum-based drugs redundant, our results suggest a specific tumour response to carboplatin when compared to cisplatin, mainly concerning the application of a NEDDylation inhibitor. In conclusion, our results indicate that preventing NEDDylation of cullins and HuR may provide an adjunct therapy to overcome tumour acquired resistance during carboplatin chemotherapy.

## Conclusion

Carboplatin is an anticancer drug that is on the World Health Organisation’s List of Essential Medicines, which categorises it as one of the most important antineoplastic drugs needed in a basic health system. It is the least toxic platinum-based drug, and is widely used in cancer treatment, especially in ovarian tumours, where it is the first-line choice of treatment. However, some patients acquire resistance to the drug during treatment, leading to clinical complications. Here, we show a potential way to sensitise carboplatin-resistant tumours through inhibition of the signalling NEDDylation pathway. Our results have implications on clinical outcomes, since we showed that inhibition of this molecular pathway in non-chemoresistant cells is not useful, and the effect was not synergistic, such as the effect previously shown with cisplatin. However, inhibition of NEDDylation was highly efficient in sensitising carboplatin-resistant cells to the drug to the same levels as those before resistance, thus allowing the continuation of this line of treatment.

## Supporting Information

S1 Table Selected mutants that presented fitness defect when exposed to the lower dose of carboplatin (250 μM). Tested by Hillenmeyer *et al*. (2008) in a chemogenomic study of more than 1000 different assay conditions. The P-value of <0.01 was chosen because, according to the authors, this was the minimum P-value to be considered as statistically significant. The entire dataset is available at http://chemogenomics.med.utoronto.ca/fitdb/fitdb.cgi.
(XLSX)

S2 Table Human homologues of yeast carboplatin-responsive genes annotated as interacting with cullins and ELAVL1 proteins and the respective reference that observed the physical interaction. (XLSX)
